# In Vitro Biological Activities and Phytochemical Analyses of *Mespilus germanica* L.

**DOI:** 10.3390/molecules31010050

**Published:** 2025-12-23

**Authors:** Ekin Kurtul, Şükran Öztürk, Selen Tekin, Özge Yılmaz, Özlem Bahadır-Acıkara

**Affiliations:** 1 Department of Pharmacognosy, Faculty of Pharmacy, Zonguldak Bülent Ecevit University, Esenköy, Zonguldak 67600, Türkiye; 2 Department of Pharmaceutical Microbiology, Faculty of Pharmacy, Zonguldak Bülent Ecevit University, Esenköy, Zonguldak 67600, Türkiye; 3 Department of Pharmacognosy, Faculty of Pharmacy, Ankara University, Yenimahalle, Ankara 06560, Türkiye

**Keywords:** anti-inflammatory activity, antimicrobial activity, bioactive foods, chlorogenic acid, epicatechin, *Mespilus germanica*

## Abstract

*Mespilus germanica* L. is one of the two species of the genus *Mespilus* L., and is distributed in several regions, including Southeastern Europe, Anatolia, the Caucasus, and parts of the Middle East. The fruits of the plant are consumed as food, and the fruits, leaves, and stem bark are traditionally used for various systemic disorders, including gastrointestinal, respiratory, urinary tract, and skin conditions, as well as menstrual irregularities. In our study, the anti-inflammatory potential and antimicrobial activities of aqueous-methanolic extracts prepared from ripe (MGRF) and unripe fruits (MGUF), leaves (MGL), and stem bark (MGB) of *M. germanica* were evaluated in vitro. Quercetin, catechin, epicatechin, ellagic acid, chlorogenic acid, and caffeic acid were analyzed using high-performance liquid chromatography. MGL exhibited the strongest activity against *Staphylococcus aureus* (MIC = 8 µg/mL), while MGB was most active against *Enterococcus faecalis* (MIC = 4 µg/mL), and fruit extracts were effective against resistant *Acinetobacter baumannii* (MIC = 16–32 µg/mL). Membrane-protective effects were more pronounced in MGUF and MGB, whereas MGL demonstrated the highest protein stabilization activity. Leaves also contained the highest levels of chlorogenic acid and epicatechin. These findings support the traditional use of *M. germanica*, though further studies are required to explore its therapeutic potential.

## 1. Introduction

*Mespilus germanica* L. (Rosaceae) is a perennial shrub or small tree that grows naturally in Southeastern Europe, Anatolia, Crimea, Caucasus, Balkan Peninsula, Greece, Northern Iran, and Northern Iraq. It is cultivated in many countries, with known cultivars including Hollandia, Nottingham, Russian, Dutch, Royal, Breda Giant, and Large Russian, and it is commonly known as medlar, muşmula, döngel, or beşbıyık [1,2]. It has been used for culinary and medicinal purposes for many years. Unripe fruits are traditionally stored between layers of hay to increase polyphenol oxidase activity, during which they turn brown, become soft, sweet, and edible [3]. Fruits are consumed raw, as well as used to make juice, jam, pickle, and cheese; the leaves are brewed and consumed as herbal tea. However, the seeds are known to be poisonous [2].

*M. germanica* has been traditionally used for therapeutic purposes in various countries. In Iran, the fruits are used as blood tonics, nerve-strengthening agents, appetite stimulants, and diuretics. They are also traditionally used to treat colitis, enteritis, diarrhea, indigestion, menstrual irregularities, and internal bleeding. Fruits are also consumed for intestinal inflammation after removing the seeds and skin, then mixed with milk, and for cholera as a soaked preparation [4]. Decoctions prepared from the fruits are used as cardiotonics, diuretics, and hypotensives, as well as for the treatment of kidney and bladder stones and constipation [5,6]. Additionally, leaves are used as a blood tonic and to treat large intestine infections, diarrhea, internal bleeding, and mouth abscesses. Moreover, soaked leaves are used for the treatment of cutaneous leishmaniasis and skin and throat disorders. Stem barks are reported to treat large intestine infections, diarrhea, and internal bleeding. They can also be used as blood tonics and diuretics. The powdered stem bark is soaked in alcohol, and the feet are washed with it to alleviate fever [4].

In Turkish folk medicine, decoctions made from *M. germanica* and *Corylus* sp. leaves are consumed to treat tuberculosis. Leaves are also used to relieve flu, bronchitis, and cough, either alone or combined with quince leaves and lemon. Infusions prepared from the leaves and dried fruits are traditionally used to treat diarrhea, asthma, and hepatitis. Additionally, branches and stem bark are employed to relieve abdominal pain, cough, and hemorrhoids. Leaves are also reported to be used for eczema, rheumatism, and diabetes. The fruits are consumed for their anti-inflammatory properties. They are used to treat dysentery, colitis, and enteritis, while infusions of the seeds serve as a diuretic for kidney and bladder stones [2,7,8,9].

*M. germanica* leaves are also used for hypoglycemia and for kidney and bladder stones in Algeria. At the same time, fresh fruits are consumed daily in Serbia to enhance eyesight, improve liver and kidney function, and act as an antidiarrheal [2].

The traditional importance of *M. germanica* has been widely reported in ethnobotanical studies, although phytochemical research and evidence for the plant’s biological activities are considerably limited. Previous studies have demonstrated the antioxidant, antimicrobial, antidiabetic, DNA-protective, cytotoxic, neuroprotective, and skin-protective properties of *M. germanica*, particularly for its leaves and fruits [2,10,11,12,13]. The biological activities of the plant are attributed to its phytochemical contents, such as flavonoids and other phenolics [12]. Furthermore, the fruits are rich in sugars, organic acids, amino acids, pectin, carotene, phenolics, and minerals. Two monoterpenoid antibiotics, genipic acid and genipinic acid, have been reported to be isolated from the fruits [2,14].

The present study aimed to support the traditional uses of *M. germanica* by evaluating its antimicrobial properties and assessing the in vitro anti-inflammatory potential of its leaves, stem bark, and both ripe and unripe fruits. The antimicrobial activities were evaluated against resistant strains of *Acinetobacter baumannii*, *A. baumannii* ATCC 19606, *Staphylococcus aureus* ATCC 29213, *Enterococcus faecalis* ATCC 29212, *Escherichia coli* ATCC 25922, colistin-resistant *E. coli* COR13946, colistin-resistant *Pseudomonas aeruginosa*, and *P. aeruginosa* ATCC 27853 using the broth microdilution method. The antimicrobial activities of the different leaf and fruit extracts were documented in previous studies. However, the present study provides the first report on the stem bark, for which no biological data have been published. Additionally, it represents the first evaluation of the plant against clinically relevant drug-resistant microorganisms, which represent an increasing global health concern. In addition, the human red blood cell membrane stabilization method and protein denaturation inhibition assay were used to examine the in vitro anti-inflammatory potential of the plant. Moreover, qualitative and quantitative analyses of quercetin, catechin, epicatechin, ellagic acid, caffeic acid, and chlorogenic acid were carried out using thin-layer chromatography (TLC) and high-performance liquid chromatography (HPLC). These compounds are well known in the literature for their roles in anti-inflammatory and antimicrobial activities and are widely distributed in the Rosaceae family.

## 2. Results

### 2.1. Extraction

Each plant material was extracted at room temperature using a methanol/water (70:30) solvent system, and the extraction yields from different parts of *M. germanica* are presented in Table 1.

### 2.2. Phytochemical Analyses

Phytochemical analyses were conducted through preliminary TLC applications, followed by qualitative and quantitative HPLC analyses. Regarding the TLC plates, quercetin, caffeic acid, and chlorogenic acid showed strong absorbance under UV_254_ nm, while catechin and epicatechin became visible after treatment with H_2_SO_4_-vanillin reagent. However, catechin and epicatechin, which have the same Rf value, as well as ellagic acid, could not be separated by normal-phase chromatography; therefore, their presence was confirmed by reverse-phase HPLC. TLC analyses showed epicatechin or catechin in leaf, stem bark, and unripe fruit extracts, while chlorogenic acid was found in leaves and unripe fruits. The ripe fruit extract did not show any bands for the tested compounds.

Qualitative HPLC analyses revealed the presence of only epicatechin and chlorogenic acid in the extracts. The HPLC chromatograms of the standard compounds are shown in the Supplementary Material. According to the quantitative HPLC analyses, the highest amounts of epicatechin (0.47) and chlorogenic acid (1.80) were found in the leaves (mg/100 g dry plant material). Chlorogenic acid was determined as 0.0032 mg/100 g dry plant material in unripe fruits. Additionally, epicatechin was found in stem bark and unripe fruits at 0.30 and 0.00124 mg/100 g dry plant material (Table 2).

**Figure 1:**
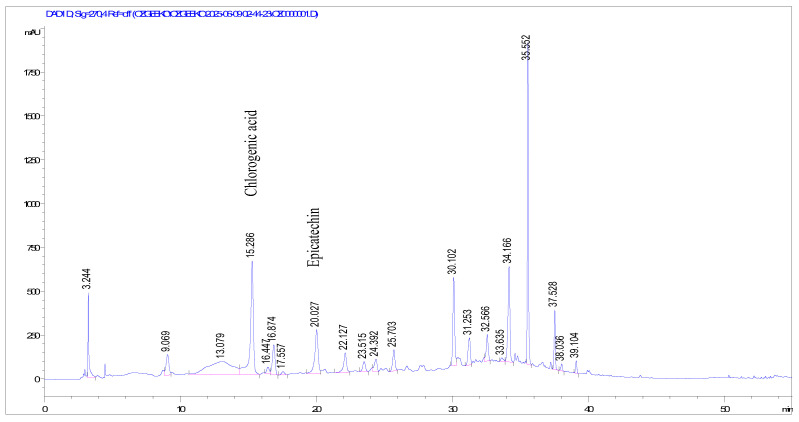
HPLC chromatogram of *Mespilus germanica* leaf extract at 270 nm with chlorogenic acid (Rt: 15.286) and epicatechin (Rt: 20.027). X-axis: Retention time (min); Y-axis: Absorbance (mAU).

**Figure 2:**
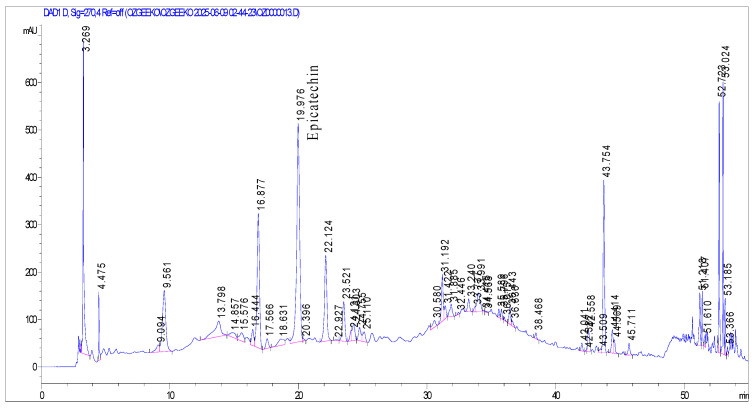
HPLC chromatogram of *Mespilus germanica* stem bark extract at 270 nm with epicatechin (Rt: 19.976). X-axis: Retention time (min); Y-axis: Absorbance (mAU).

**Figure 3:**
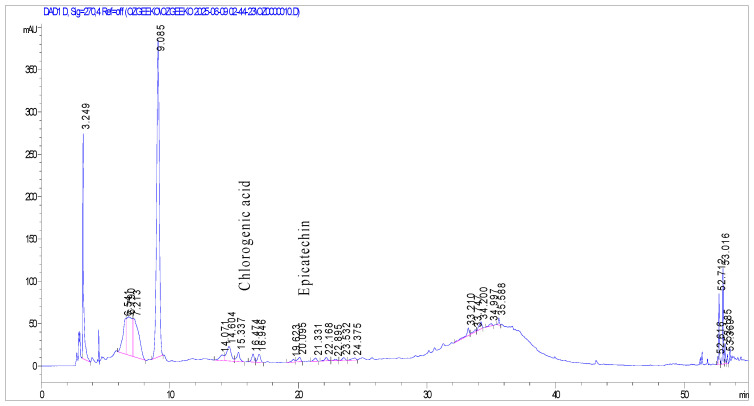
HPLC chromatogram of *Mespilus germanica* unripe fruit extract at 270 nm with chlorogenic acid (Rt: 15.337) and epicatechin (Rt: 20.095). X-axis: Retention time (min); Y-axis: Absorbance (mAU).

The LOD and LOQ values were found to be 0.21 and 0.70 µg/mL for catechin; 0.06 and 0.20 µg/mL for chlorogenic acid; 0.25 and 0.75 µg/mL for caffeic acid; 0.15 and 0.50 µg/mL for epicatechin; 0.24 and 0.80 µg/mL for ellagic acid; and 0.60 and 0.18 µg/mL for quercetin, respectively. None of the tested compounds was detected in the ripe fruit extract. The HPLC chromatograms of the extracts with the detected compounds are shown in Figure 1, Figure 2 and Figure 3.

### 2.3. Antimicrobial Activity

The bacteriostatic activity of the extracts against standard and clinical bacterial strains was evaluated as MIC values according to the recommendations of the European Committee on Antimicrobial Susceptibility Testing (EUCAST, 2019) [15], as shown in Table 3.

The leaf extract (MGL) showed similar activity to MGB, with MIC values of 8–64 µg/mL for ATCC strains and 32–64 µg/mL for the resistant clinical isolates. The unripe fruit extract (MGUF) and ripe fruit extract (MGRF) of the plant displayed relatively weak activity against the ATCC strains, with MIC values ranging from 16 to 128 µg/mL, whereas MIC values of 32 µg/mL indicated significant antimicrobial activity against multidrug-resistant (MDR) and pandrug-resistant (PDR) strains.

### 2.4. Anti-Inflammatory Activity

#### 2.4.1. Human Red Blood Cell (HRBC) Membrane Stabilization

The highest activity in the HRBC membrane stabilization test was observed in MGUF with an IC_50_ value of 1011.87 µg/mL, followed by MGB (IC_50_ = 1300.43 µg/mL). MGL and MGRF were not able to inhibit hemolysis by 50%, even at high concentrations. MGB inhibited the heat-induced hemolysis dose-dependently in the range of 800–1400 µg/mL, while MGL displayed linear inhibition between 800 and 1200 µg/mL. MGL showed the highest stabilization (41%) at a concentration of 1200 µg/mL, whereas MGRF exhibited 33% stabilization at 1400 µg/mL. Higher concentrations of MGUF and MGRF resulted in reduced activity. None of the extracts showed inhibition below 800 µg/mL. Diclofenac sodium (DFS), used as a positive control, reached 58.03% inhibition at 100 µg/mL and showed 17.26% inhibition at 25 µg/mL. Notably, although the observed effects were obtained at relatively high concentrations, the HRBC stabilization activities of MGUF at 1200 and 1400 µg/mL and MGB at 1400 µg/mL were statistically comparable to 100 µg/mL DFS, providing a preliminary indication of membrane-stabilizing activity in this in vitro model (Table 4).

#### 2.4.2. Protein Denaturation

The results of the bovine serum albumin (BSA) denaturation analyses are shown in Table 5.

According to our findings, DFS showed dose-dependent activity at concentrations of 25, 50, and 100 µg/mL with an IC_50_ value of 34.70 µg/mL. MGB and MGL demonstrated significant activity in the range of 50–1000 µg/mL in a dose-dependent manner; however, at concentrations above 1000 µg/mL, protein denaturation was induced. Additionally, MGUF and MGRF were inactive at low doses. MGUF induced the denaturation above 1000 µg/mL, and MGRF above 2000 µg/mL. The IC_50_ values of MGB, MGL, MGUF, and MGRF were established as 499.58, 491.63, 783.21, and 1120.34 µg/mL, respectively. These findings indicate that stem bark and leaf extracts showed relatively higher protein denaturation inhibition compared to the fruit extracts; however, these effects were observed at comparatively high IC_50_ values and concentrations. Furthermore, all extracts exhibited protein stabilization activity only at their highest tested concentrations, which were statistically comparable to that of DFS at 100 µg/mL in this in vitro assay.

## 3. Discussion

Medicinal plants have been used since ancient times and are still widely used today because of their lower side effect profile compared to conventional drugs [12,16]. Natural products obtained from medicinal plants are recognized as significant contributors to the discovery and development of new pharmaceuticals. It is known that about 25% of currently approved pharmaceuticals include compounds derived from higher plants. According to the World Health Organization, plants are commonly used for primary healthcare purposes, especially in developing countries.

In recent years, the antimicrobial potential of medicinal plants has attracted increasing attention. Resistance easily develops against synthetic and semi-synthetic antibacterial drugs. Additionally, these antibiotics may cause side effects such as hypersensitivity and immunosuppression. Considering these adverse effects, there is a growing need to develop new antimicrobial agents with lower toxicity to the host organism [17]. Inflammation is an immune system response to various stimuli, including infections, which can progress to chronic inflammation [18,19]. Chronic inflammation has been linked to systemic disorders such as diabetes, cardiovascular diseases [20], hypertension [21], arthritis [22], osteoarthritis [23], Alzheimer’s disease [24], and cancer [25]. Non-steroidal anti-inflammatory drugs (NSAIDs), metformin, statins, and corticosteroids are commonly used to control inflammatory responses, which can cause more severe complications such as gastrointestinal disorders, kidney dysfunction, or liver dysfunction. Due to their anti-inflammatory compounds, natural products are considered promising candidates for managing inflammation with lower toxicity and fewer side effects [26]. Previous studies have shown that phenolic compounds in medicinal plants exhibit antioxidant, anticancer, neuroprotective, anti-inflammatory, and antimicrobial activities. In particular, phenolic acids and flavonoids are reported to have strong antimicrobial and anti-inflammatory effects [13,27,28,29,30].

*Mespilus germanica*, one of the two species of the genus, has various traditional uses and edible fruits. In light of this information, our study investigated the antimicrobial activity of the leaves, stem bark, and fruits, together with their phytochemical composition, and evaluated membrane-stabilizing and protein denaturation inhibitory effects as in vitro indicators related to inflammation.

Previous phytochemical studies on *M. germanica* have mostly focused on the fruits and leaves. The phenolic acid, caffeic acid, was found in unripe, semi-ripe, and ripe fruits at concentrations of 79.8, 26.8, and 30.1 µg/g, respectively. Catechin was detected at 26.3, 17.95, and 20.4 µg/g, while epicatechin was present at 26.65, 14.4, and 20.25 µg/g. Additionally, quercetin was found at concentrations of 1.95, 0.6, and 0.4 µg/g in unripe, semi-ripe, and ripe fruits, respectively. The total phenolic content was reported to increase from 2.98 mg GAE/100 g to 28.7 mg GAE/100 g during ripening [31].

UPLC analysis of the 75% ethanolic extract of ripe fruits revealed the presence of chlorogenic acid, caffeic acid, and quercetin at concentrations of 78.81, 8.55, and 2.00 mg/kg dry weight, respectively, [32]. Zolnierczyk et al. [13] reported the highest polyphenolic content in the 80% ethanolic extract of unripe fruit skins (1148 mg GAE/100 g dry weight), compared to unripe fruit pulp, ripe fruit pulp, and ripe fruit skin. Furthermore, the fruits were also found to contain protocatechuic acid, syringic acid, vanillic acid, ferulic acid, hesperidin, and epigallocatechin. The leaves of *M. germanica* have been reported to contain benzoic acid, p-hydroxybenzoic acid, vanillic acid, caffeic acid, ferulic acid, sinapic acid, p-coumaric acid, and hesperidin [33]. The ethanolic extract of leaves was also found to contain 189.22 mg GAE/g total polyphenols and 57.55 mg QE/g total flavonoids [34].

The phenolic composition of *M. germanica* was found to be dependent on both the specific plant part and the maturity of the fruits, as revealed by our phytochemical analyses. Considering the TLC and HPLC results together, epicatechin and chlorogenic acid were found at relatively higher levels in the leaves, whereas these compounds were not detected even at low concentrations in the ripe fruit extract. This pattern may be associated with the degradation, transformation, or polymerization of phenolics during the maturation process. Our findings also revealed differences in the amounts of caffeic acid, catechin, epicatechin, and chlorogenic acid compared with previous studies, particularly for the ripe fruit samples. Such phytochemical variations may result from differences in extraction solvents and techniques, collection region, and ripening parameters among studies. In contrast to previous reports on other plant parts, quercetin, catechin, and caffeic acid were not detected in any of our samples, while chlorogenic acid and epicatechin were consistently present. Moreover, the relatively lower polyphenolic content of the extracts indicates the sensitivity of the plant’s chemical composition to environmental conditions. The chromatograms also showed several other prominent peaks, suggesting the presence of additional, currently unidentified compounds that may contribute to the observed activities.

The antimicrobial activity of *M. germanica* has been evaluated in a limited number of studies previously. The MIC values of ethanolic and methanolic leaf extracts against *P. aeruginosa*, *S. aureus*, and *E. coli* were reported as 125 mg/mL by Ahmady-Abschin et al. [35]. The leaf extract prepared with 70% acetone showed significant bactericidal activity against *Klebsiella pneumoniae* [16]. The ethanolic extract of the leaves demonstrated a remarkable therapeutic effect on lesions in Leishmania-infected mice [36]. Methanolic (70%) extract of fruits was found to be active on *B. subtilis*, *B. cereus*, *P. aeruginosa*, *E. faecalis,* with a MIC value of 32 mg/mL, while the MIC value was determined as 64 mg/mL for *S. epidermidis*, *S. aureus*, *K. pneumoniae*, *E. coli*, *S. salivarius*, *S. mutans*, *P. aeruginosa,* and *S. typhimurium* [12].

Denizkara et al. [37] tested fruit extracts of different polarities collected in November and demonstrated that the aqueous extract exhibited the highest antimicrobial activity against *S. aureus* and *Penicillium chrysogenum*.

In the present study, the leaf and stem bark extracts exhibited markedly stronger activity, particularly against Gram-negative and drug-resistant strains, with MIC values ranging from 4 to 64 µg/mL. These findings indicate that these extracts possess a noticeably higher antimicrobial potential compared with previous reports. The antimicrobial activity of the fruit extracts was generally consistent with earlier studies; however, the MIC values of 16–32 µg/mL observed against MDR and PDR *A. baumannii* point to a stronger effect. These differences may be attributed to environmental conditions, extraction parameters, and variations in plant material.

The membrane-stabilizing and protein-denaturation-inhibitory activities of the extracts were evaluated using HRBC membrane stabilization and BSA stabilization assays in our study. Baradaran et al. [38] tested the anti-inflammatory activity of the fruits of *M. germanica* collected from Iran and found significant inhibition in formalin-induced rats, particularly during the chronic phase. That was the only reported anti-inflammatory finding for *M. germanica*. However, numerous studies have demonstrated the anti-inflammatory and antimicrobial activities of chlorogenic acid and epicatechin, which were also detected in our active extracts.

Chlorogenic acid reversed the reduction in collagen II and the increase in iNOS/NO, IL-6, MMP-13, and COX-2/PGE2 levels in IL-1β-induced human SW-1353 chondrocytes, an in vitro model of osteoarthritis [39]. In a rat model of spinal cord injury (SCI), it also reduced inflammation, nitric oxide synthase activity, and COX-2 expression [40]. Chlorogenic acid inhibited the nuclear translocation of NF-κB and reduced the expression of NO, COX-2, iNOS, and Ninjurin-1 in LPS-induced mice RAW264.7 macrophages and BV2 microglia cells [41]. Chlorogenic acid and epicatechin were tested in mice with dextran sulfate-induced colitis. Chlorogenic acid suppressed the expression of colonic macrophage inflammatory protein-2 and IL-1β, while epicatechin reduced the levels of TNF-α, IL-6, NO, MPO, and MDA. Chlorogenic acid also inhibited TNF-α and H_2_O_2_-induced IL-8 production in human Caco-2 colon cell lines [42,43]. Epicatechin also downregulated COX-2, enhanced cell proliferation in rats with colitis, and suppressed pro-inflammatory mediators in LPS-stimulated RAW264.7 macrophages [44,45].

Antimicrobial activities of chlorogenic acid and epicatechin have been well documented in previous studies. Lou et al. [46] reported MIC values of 40 and 80 µg/mL for chlorogenic acid against *S. aureus* and *E. coli*, respectively. Li et al. [47] showed that it inhibited *S. aureus* proliferation by disrupting the bacterial cell membrane. Chlorogenic acid and epicatechin displayed strong antibacterial activity against *Bacillus* spp., *Clostridium* spp., and *Listeria* spp., which are known as foodborne pathogens [48]. Epicatechin isolated from *Smilax glabra* Roxb. (Liliaceae) exhibited antimicrobial effects on MRSA, *S. aureus*, and, with MIC values, *E. faecalis* measured at 1.03, 0.517, and 1.03 mM [49]. Furthermore, MIC values ranging from 12.5 to 100 μg/mL were reported for epicatechin from *Camellia sinensis* L. against several pathogens, including *S. aureus*, *S. pyogenes*, *E. coli*, *P. aeruginosa*, and *Enterobacter cloacae* [50]. Moreover, epicatechin has been shown to inhibit antibiotic efflux pumps in *S. aureus* strains resistant to penicillin, erythromycin, methicillin, and clindamycin, with MIC values ranging from 32 to 512 μg/mL [51].

When the biological activity and phytochemical analysis results were considered together, it was observed that the stem bark extract, which had the highest epicatechin content, showed the strongest activity against *E. faecalis*, while the leaf extract, with the highest chlorogenic acid content, exhibited the strongest activity against *S. aureus*, consistent with previously reported findings. The leaf extract also had the lowest IC_50_ in BSA stabilization, likely due to its high chlorogenic acid content. Notably, strong erythrocyte membrane stabilization was also observed for unripe fruits and stem bark, both rich in epicatechin.

In conclusion, each extract exhibited membrane-stabilizing and protein denaturation inhibitory effects through different in vitro assays, while significant antimicrobial effects were observed against specific bacterial groups. Although chlorogenic acid and epicatechin may partly contribute to the observed activities, the presence of other prominent unidentified constituents suggests that additional compounds are likely involved, and the biological effects of the extracts cannot be fully attributed to previously reported phenolics. These findings highlight the need for advanced molecular studies and in vivo testing on *M. germanica*, as well as time-kill and bactericidal assays to further clarify its antimicrobial properties. Isolating bioactive compounds from *M. germanica* and evaluating their pharmacological properties may contribute to the discovery of novel therapeutic agents. Standardized extracts from different parts of the plant could serve as promising treatments for resistant infections, while the dose-dependent membrane stabilization and protein denaturation effects were observed only at relatively high concentrations and therefore are not sufficient to support anti-inflammatory activity. The traditional use of the plant for various ailments over many years, particularly in ways consistent with the biological activities demonstrated in our study, also supports its consideration as a traditional herbal medicinal product.

## 4. Materials and Methods

### 4.1. Plant Material

The plant materials were collected from Uludağ, Bursa, Türkiye, in 2023 and were identified by Assoc. Prof. Dr. Ayşe Kaplan from the Faculty of Science, Zonguldak Bülent Ecevit University. Leaves, stem bark, and unripe fruits were collected in August, while the ripe fruits were collected in November. The voucher specimens are deposited in Ankara University, Faculty of Pharmacy Herbarium (AEF 31127). Leaves were dried at room temperature. Unripe fruits were lyophilized (Christ Alpha 1-2 LD Plus lyophilizer, Martin Christ GmbH, Osterode am Harz, Germany), after removing the seeds to inhibit polyphenol oxidase activity. Ripe fruits were dried at room temperature after removing the seeds. The dried plant materials were powdered and extracted separately with 70% methanol. Plant materials were macerated in the solvent mixture for three days, then filtered. The maceration procedure was repeated five times for each extract, and the filtrates were combined and evaporated (BUCHI Rotary Evaporator, BUCHI Labortechnik AG, Flawil, Switzerland) under vacuum at 38–40 °C.

### 4.2. Phytochemical Analyses

Thin-layer chromatography was used for preliminary qualitative analysis. Standard compounds—quercetin (Sigma-Aldrich, St. Louis, MO, USA), ellagic acid (TCI, Tokyo Chemical Industry), chlorogenic acid (TCI), caffeic acid (TCI), catechin (TCI), and epicatechin (TCI)—and extracts were applied on pre-coated silica gel plates (Merck, Darmstadt, Germany; product no. 1.05554). The plates were developed in a mobile phase consisting of a mixture of ethyl acetate, methanol, and water (80:13.5:10, *v*/*v*/*v*), which provided the best separation for the compounds and extracts. The plates were examined under 254 and 366 nm UV light (UV lamp, model UVGL-58, supplied by Neolab, Heidelberg, Germany), then treated with 10% ethanolic sulfuric acid solution and 1% ethanolic vanillin solution, followed by heating at 100 °C.

Qualitative HPLC analyses (Agilent 1200 HPLC system, UV-DAD detector, Agilent Technologies, Waldbronn, Germany), were performed to confirm the presence of compounds—ellagic acid, catechin, and epicatechin—that could not be separated by normal-phase chromatography.

Quantitative analyses of the detected compounds were carried out by HPLC on a C18 column (ACE, 250 × 4.6 mm, 5 µm). The mobile phase consisted of water containing 0.2% o-phosphoric acid (A), acetonitrile (B), and methanol (C). The flow was initiated with a mixture of A/B (90:10) at a flow rate of 1 mL/min, and the mixture was changed to A/B (89.6:10.4) at minute 5. At the same time, the flow rate was reduced to 0.5 mL/min. The solvent composition was adjusted to A/B (89.4:10.6) at minute 15, and the flow rate was increased to 1 mL/min. At minute 22, the flow rate was raised to 1.2 mL/min. At minute 25, the solvent ratio was changed to A/B (85:15) and maintained for 5 min. The flow rate was reduced to 1 mL/min at minute 30, and the solvent composition was changed to A/B (57:43) at minute 45. Finally, 100% solvent C was used during the last 5 min. The column temperature was 40 °C, and the injection volume was 10 µL. The total analysis time was 55 min, with an additional 5 min post-run time. HPLC chromatograms were examined at 254, 270, and 330 nm wavelengths.

The extracts were dissolved in methanol at a concentration of 100 mg/mL and injected in triplicate. The standard compounds epicatechin and chlorogenic acid, which were qualitatively detected in the extracts, were injected into the system in triplicate at concentrations of 1, 0.5, 0.25, 0.1, 0.05, 0.025, 0.01, and 0.005 mg/mL. Calibration curves were made up by plotting the peak areas against the corresponding concentrations. The amounts of the compounds in the extracts and in the dry plant materials were calculated using the calibration equations. Limit of detection (LOD) and Limit of Quantification (LOQ) values of the standard compounds were established at signal/noise ratios of 3 and 10, respectively.

### 4.3. Antimicrobial Activity

#### 4.3.1. Strains

Three multidrug-resistant and three pandrug-resistant *A. baumannii* strains, as well as *A. baumannii* ATCC 19606, *S. aureus* ATCC 29213, *E. faecalis* ATCC 29212, *E. coli* ATCC 25922, colistin-resistant *E. coli* COR13946, colistin-resistant *P. aeruginosa*, and *P. aeruginosa* ATCC 27853 standard strains were included in the study. The clinical isolates were selected from strains that were routinely isolated in the hospital and preserved in the ZBEUN Faculty of Pharmacy culture collection. The strains were passaged onto Mueller-Hinton Agar (MHA) medium from the −80 °C freezer in the Laboratory of Pharmacology, Faculty of Medicine, Zonguldak Bülent Ecevit University. The bacterial inocula were incubated at 35 °C for 18–24 h. The strains were activated twice before use in the study.

#### 4.3.2. Antimicrobial Susceptibility Test

Antimicrobial activity was evaluated using the microdilution assay, based on the ISO 2006 recommendations of the EUCAST [15]. Microorganisms were inoculated into Mueller-Hinton Broth (MHB) medium and incubated at 35 °C until reaching the logarithmic phase. Each bacterial inoculum was adjusted to the standard 0.5 McFarland turbidity (1.5 × 10^8^ CFU/mL) using a densitometer. The suspensions were diluted to 106 CFU/mL with MHB. Cefepime and colistin were used as standard drugs. Two-fold serial dilutions of the compounds and extracts were prepared in cation-adjusted Mueller-Hinton Broth (MHB) in microplates. All extracts and the standard compounds were dissolved in dimethyl sulfoxide (DMSO). The concentration range of the extracts was set from 128 to 0.25 μg/mL, while colistin was prepared at 64 to 0.125 μg/mL and cefepime at 64 to 0.0625 μg/mL. The bacterial suspensions were prepared in microplates at a concentration of 5 × 10^5^ CFU/mL and incubated at 35 °C ± 1 °C for 18–20 h. The lowest concentration that inhibited bacterial growth was determined as the minimum inhibitory concentration (MIC). Each test was carried out in triplicate. Two wells on each microplate were incubated with bacterial inoculum without extract as growth controls, and two wells containing only sterile broth were incubated on each plate as sterility controls.

A solvent control containing DMSO at the highest concentration used in the test wells was included, and no inhibitory effect of DMSO was observed under these experimental conditions.

### 4.4. Anti-Inflammatory Activity

#### 4.4.1. Human Red Blood Cell (HRBC) Membrane Stabilization Assay

The HRBC membrane stabilization assay was conducted according to the method described by Kurtul et al. [52], with minor modifications (Ethical approval was obtained from the ZBEUN Non-Interventional Clinical Research Ethics Committee on 7 February 2024, with approval number 2024/3). 5 mL of blood was collected into lithium heparinized tubes from individuals who had not taken any non-steroidal anti-inflammatory, steroidal, or anticoagulant drugs for at least four weeks. The blood samples were stored at 4 °C for 24 h and then centrifuged at 3000 rpm for 10 min. The pellet was washed with phosphate-buffered isosaline (0.09%, pH 7.2) (PBS) three times, and then suspended in PBS to obtain a 10% suspension. Each extract was dissolved in PBS at concentrations ranging from 100 to 1600 µg/mL. The reaction mixture consisted of 1 mL of erythrocyte suspension and 1 mL of extract. Diclofenac sodium (Sigma-Aldrich) at concentrations of 25, 50, and 100 µg/mL was used as the positive control, while PBS served as the negative control. The mixtures were vortexed and incubated at 56 °C for 30 min. After cooling to room temperature, the mixtures were centrifuged at 5000 rpm for 10 min. The absorbance of the supernatants was measured at 560 nm using a microplate reader (Thermo Scientific, Thermo Fisher Scientific Inc., Waltham, MA, USA). The experiment was carried out in triplicate. The percentage inhibition values of each test material were calculated using the following equation:

*Stabilization* (%) = (*Absorbance of control* − *Absorbance of test material*)/*Absorbance of control* × *100*

#### 4.4.2. Protein Denaturation Assay

The protein denaturation assay was carried out according to the method described by Aidoo et al. [53] with minor modifications. 3% albumin solution was prepared by dissolving 3 g of bovine serum albumin (BSA) in 100 mL of PBS (pH 6.4). The extracts were dissolved in PBS at concentrations ranging from 50 to 2000 µg/mL. Then, 0.5 mL of BSA solution and 0.5 mL of extract were thoroughly mixed by vortexing and incubated at 37 °C for 15 min. After incubation, the tubes were placed in a 60 °C water bath for 5 min. The mixtures were cooled to room temperature, and their turbidity was measured at 660 nm using a UV spectrophotometer. The negative control consisted of 0.5 mL of BSA solution and 0.5 mL of PBS, while diclofenac sodium was used as the positive control at concentrations of 25, 50, and 100 µg/mL. The experiment was carried out in triplicate. The percentage inhibition values of each test material were calculated using the following equation:

*Inhibition* (%) = (*Absorbance of control* − *Absorbance of test material*)/*Absorbance of control* × *100*

#### 4.4.3. Statistical Analyses

Statistical analyses were performed using SPSS version 16.0 (IBM Corp., Armonk, NY, USA). One-way ANOVA followed by Tukey’s HSD post hoc test was used to determine statistical significance. *p* < 0.05 was considered statistically significant.

## Figures and Tables

**Table 1:** Extract weight and extraction yield of different parts of *Mespilus germanica*.

The Parts of the Plant	Dry Weight of Plant Material (g)	Extract Weight (g)	Extraction Yield (%)
Leave	167.26	75.57	45.18
Stem bark	151.88	12.73	8.38
Unripe fruit	212.92	32.73	15.38
Ripe fruit	174.68	49.09	28.10

**Table 2:** Quantification of chlorogenic acid and epicatechin in extracts (µg/mg) and dry plant materials (mg/100 g).

	Leaf		Stem Bark		Unripe Fruit	
	Extract (µg/mg)	Dry Plant Material (mg/100 g)	Extract (µg/mg)	Dry Plant Material (mg/100 g)	Extract (µg/mg)	Dry Plant Material (mg/100 g)
**Epicatechin**	10.4965 ± 0.1128	0.4743 ± 0.0051	35.9395 ± 0.3519	0.3011 ± 0.0030	0.0809 ± 0.0029	0.00124 ± 0.00004
**Chlorogenic acid**	39.8006 ± 0.0986	1.7983 ± 0.0045	-	-	0.2087 ± 0.0005	0.0032 ± 0.0000

-: not detected.

**Table 3:** MIC values of the extracts.

Minimum Inhibitory Concentrations (MIC-µg/mL)													
	*E. coli* ATCC 25922	*E. coli* COR 13946	*A. baumannii* ATCC 19606	*E. faecalis* ATCC 29212	*S. aureus* ATCC 29213	*P. aeruginosa* ATCC 27853	*P. aeruginosa* COR	*MDR1*	*MDR2*	*MDR3*	*PDR1*	*PDR2*	*PDR3*
MGB	16	16	64	4	16	16	16	64	64	64	32	64	64
MGL	64	64	16	32	8	32	32	64	32	32	32	64	64
MGUF	64	64	16	64	32	64	64	16	32	32	32	32	32
MGRF	64	64	16	128	>128	32	32	32	32	32	32	32	32
CO	0.0625	0.0625	0.0625	0.0625	0.0625	0.0625	8	2	8	8	8	32	64
CEF	0.125	2	0.125	1	2	1	2	2	4	16	16	32	32

**MDR1**; **MDR2**; **MDR3**: Multidrug-resistant *Acinetobacter baumannii* clinical isolates; **PDR1**; **PDR2**; **PDR3**: Pandrug-resistant *Acinetobacter baumannii* clinical isolates; **MGB**: *Mespilus germanica* stem bark extract; **MGL**: *Mespilus germanica* leaf extract; **MGUF**: *Mespilus germanica* unripe fruit extract; **MGRF**: *Mespilus germanica* ripe fruit extract; **COR**: Colistin-resistant isolate; **CO**: Colistin; **CEF**: Cefepime.

**Table 4:** HRBC membrane stabilization activities.

	Stabilization (%)				
Concentration (µg/mL)	MGL	MGB	MGUF	MGRF	DFS
25	-	-	-	-	17.26 ^a,b,c^
50	-	-	-	-	48.73 ^h,i,j,k,l^
100	-	-	-	-	58.03 ^k,l,m^
800	30.52 ^c,d,e,f,g^	9.75 ^a^	40.15 ^f,g,h,i,j^	-	-
1000	37.52 ^e,f,g,h,i^	34.26 ^e,f,g,h^	49.39 ^i,j,k,l^	19.42 ^a,b,c,d^	-
1200	41.75 ^f,g,h,i,j^	43.92 ^g,h,i,j^	60.60 ^l,m^	24.06 ^a,b,c,d,e^	-
1400	30.19 ^a,b^	54.48 ^j,k,l,m^	65.03 ^m^	33.00 ^d,e,f,g^	-
1600	28.45 ^b,c,d,e,f^	25.02 ^b,c,d,e^	-	-	-
	**IC_50_ ± SEM (µg/mL)**				
IC_50_ (µg/mL)	-	1300.43 ± 10.60	1011.868 ± 30.39	-	75.91 ± 1.60

**MGL**: *Mespilus germanica* leaf extract; **MGB**: *Mespilus germanica* stem bark extract; **MGUF**: *Mespilus germanica* unripe fruit extract; **MGRF**: *Mespilus germanica* ripe fruit extract; **DFS**: Diclofenac sodium; One-way ANOVA followed by post hoc Tukey HSD test. ^a–m^: Different letters indicate statistically significant differences between groups (*p* < 0.05).

**Table 5:** BSA stabilization activities.

	Stabilization (%)				
Concentration (µg/mL)	MGL	MGB	MGUF	MGRF	DFS
25	-	-	-	-	30.40 ^f^
50	5.52 ^a,b^	4.99 ^a^	-	-	75.98 ^j^
100	13.00 ^c,d^	12.63 ^b,c,d^	-	-	99.20 ^l^
250	21.04 ^e^	21.95 ^e^	-	-	-
500	58.70 ^i^	59.70 ^i^	8.00 ^a,b,c^	-	-
600	-	-	16.56 ^d,e^	-	-
700	-	-	30.02 ^f^	-	-
800	-	-	40.60 ^g^	-	-
900	-	-	74.74 ^j^	-	-
1000	97.06 ^l^	93.74 ^k,l^	94.90 ^k,l^	41.31 ^g^	-
1200	-	-	-	49.11 ^h^	-
1400	-	-	-	70.67 ^j^	-
1600	-	-	-	87.85 ^k^	-
1800	-	-	-	95.36 ^l^	-
2000	-	-	-	96.86 ^l^	-
	**IC_50_ ± SEM (µg/mL)**				
IC_50_ (µg/mL)	491.63 ± 0.02	499.58 ± 0.02	783.21 ± 3.57	1120.34 ± 0.84	34.70 ± 1.50

**MGL**: *Mespilus germanica* leaf extract; **MGB**: *Mespilus germanica* stem bark extract; **MGUF**: *Mespilus germanica* unripe fruit extract; **MGRF**: *Mespilus germanica* ripe fruit extract; **DFS**: Diclofenac sodium; One-way ANOVA followed by post hoc Tukey HSD test. ^a–l^: Different letters indicate statistically significant differences between groups (*p* < 0.05).

## Data Availability

All data generated or analyzed during this study are included in this article.

## Supplementary Materials

The following supporting information can be downloaded at https://www.mdpi.com/article/10.3390/molecules31010050/s1. Supplementary Material: Figure S1: HPLC chromatograms of standard compounds. A: 254 nm, B: 270 nm, C: 330 nm.

## Abbreviations

The following abbreviations are used in this manuscript:

DMSO	Dimethyl sulfoxide
MDR	Multidrug resistant
PDR	Pandrug resistant
COR	Colistin-resistant isolate
CO	Colistin
CEF	Cefepime
DFS	Diclofenac sodium
MGL	*Mespilus germanica* leaf extract
MGB	*Mespilus germanica* stem bark extract
MGUF	*Mespilus germanica* unripe fruit extract
MGRF	*Mespilus germanica* ripe fruit extract
