# Supplementary material for: In Vitro Biological Activities and Phytochemical Analyses of Mespilus germanica L
Source: Molecules. 2025 Dec 23;31(1):50. doi: 10.3390/molecules31010050 (PMC12786780; doi:10.3390/molecules31010050)
Supplement: Supplementary file 1 [file molecules-31-00050-s001.zip › molecules-4021590-supplementary.pdf]

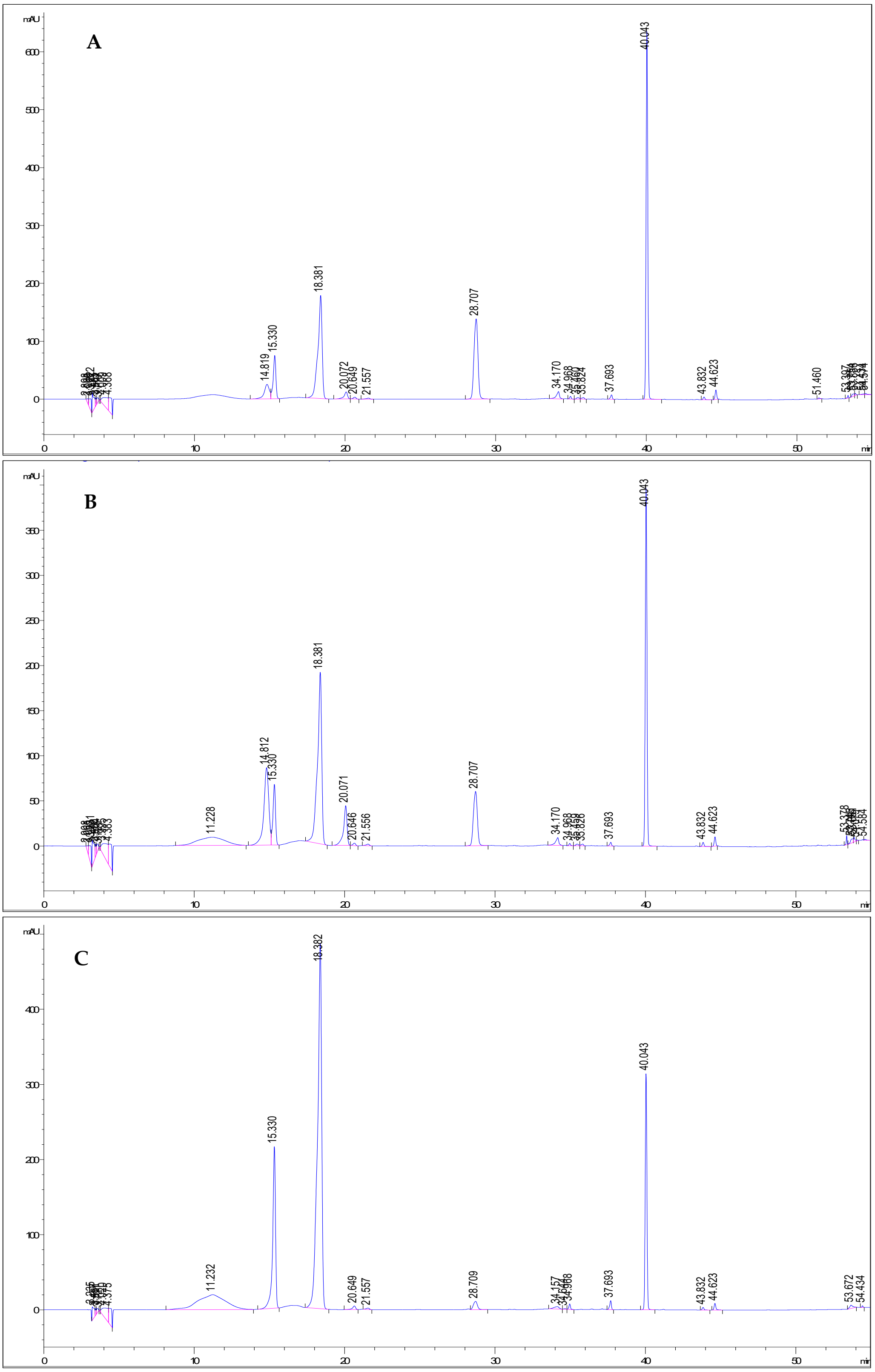

Supplementary material. HPLC chromatograms of standard compounds. **A:** 254 nm, **B:** 270 nm, **C:** 330 nm

Retention times: catechin-14.812 min (270 nm), chlorogenic acid-15.330 min (270 and 330 nm), caffeic acid-18.381 min, epicatechin-20.071 min (270 nm), ellagic acid-28.707 min (254 nm), and quercetin-40.043 min. X-axis: Retention time (min); Y-axis: Absorbance (mAU).
